# Occupational conditions and the risk of the use of amphetamines by truck drivers

**DOI:** 10.1590/S0034-8910.2015049005944

**Published:** 2015-09-29

**Authors:** Lúcio Garcia de Oliveira, Letícia Maria de Araújo de Souza, Lúcia Pereira Barroso, Marcela Júlio César Gouvêa, Carlos Vinícius Dias de Almeida, Daniel Romero Muñoz, Vilma Leyton

**Affiliations:** I Departamento de Medicina Legal, Ética Médica, Medicina Social e do Trabalho. Faculdade de Medicina. Universidade de São Paulo. São Paulo, SP, Brasil; II Departamento de Estatística. Instituto de Matemática e Estatística. Universidade de São Paulo. São Paulo, SP, Brasil

**Keywords:** Transportation, manpower, Amphetamines, Working Conditions, Risk Factors, Occupational Risks, Occupational Health, Cross-Sectional Studies

## Abstract

**OBJECTIVE:**

To test whether the occupational conditions of professional truck drivers are associated with amphetamine use after demographic characteristics and ones regarding mental health and drug use are controlled for.

**METHODS:**

Cross-sectional study, with a non-probabilistic sample of 684 male truck drivers, which was collected in three highways in Sao Paulo between years 2012 and 2013. Demographic and occupational information was collected, as well as data on drug use and mental health (sleep quality, emotional stress, and psychiatric disorders). A logistic regression model was developed to identify factors associated with amphetamine use. Odds ratio (OR; 95%CI) was defined as the measure for association. The significance level was established as p < 0.05.

**RESULTS:**

The studied sample was found to have an average age of 36.7 (SD = 7.8) years, as well as low education (8.6 [SD = 2.3] years); 29.0% of drivers reported having used amphetamines within the twelve months prior to their interviews. After demographic and occupational variables had been controlled for, the factors which indicated amphetamine use among truck drivers were the following: being younger than 38 years (OR = 3.69), having spent less than nine years at school (OR = 1.76), being autonomous (OR = 1.65), working night shifts or irregular schedules (OR = 2.05), working over 12 hours daily (OR = 2.14), and drinking alcohol (OR = 1.74).

**CONCLUSIONS:**

Occupational aspects are closely related to amphetamine use among truck drivers, which reinforces the importance of closely following the application of law (Resting Act (“Lei do Descanso”); Law 12,619/2012) which regulates the workload and hours of those professionals. Our results show the need for increased strictness on the trade and prescription of amphetamines in Brazil.

## INTRODUCTION

On October 6, 2011, Brazilian Health Surveillance Agency (ANVISA), by means of Resolution 52 (RDC 52/2011) from its Collegiate Board of Directors, has prohibited the production, trade, prescription, and consequently, amfepramone, fenproporex, and mazindol consumption.^a^ Those substances are known as amphetamine derivatives, amphetamines, stimulants, and anorectics – the last two having those names due to their effects in the central nervous system. Despite that, such substances are still being consumed by truck drivers, possibly to the same extent as they were being consumed before the aforesaid regulation.^14^

In this context, poor working conditions have been encouraging truck drivers to use amphetamines;^4^ ^-^ ^6^ especially nowadays, when the road transportation industry has become increasingly competitive.^2^ ^,^ ^22^ Thus, amphetamines have been used to reduce fatigue and to preserve focus and attention while at work.^6^ ^,^ ^9^ ^,^ ^15^

A systematic literature review^6^ showed that amphetamines rank second on the list of the most used drugs among truck drivers (being only second to alcoholic beverages). This study showed that 29.5% of drivers reported having used amphetamines, whereas 8.5% were found to have traces of those substances when biological samples from them were submitted to toxicology screens.^6^

Confirming the reasons which had already been mentioned by truck drivers, amphetamines can improve attention.^15^ However, increased blood plasma concentrations of those substances impairs driving performance,^13^ due to euphoria, quick flow of ideas, increased mental skill sensation, and even paranoid though, hallucinations, and mental confusion experienced by users.^23^ Collectively, those effects hinder motor coordination, concentration, and judgment – which are required whilst driving any vehicles.^23^ Thus, use of amphetamines or other psychotropic substances does not befit safe driving.^17^ ^,^ ^23^

In this scenario, amphetamines are still the most frequently found substances in biological samples from drivers who are pulled over due to bad driving performance.^8^ They can also be found in biological samples from fatally-wounded drivers,^3^ ^,^ ^5^ ^,^ ^10^ and its use is associated with traffic accidents,^4^ thus jeopardizing the safety and health of those drivers and the wellness of the population in general.^19^

As a response to the amphetamine use problem, the United Nations (UN) suggest the most opportune and accurate control strategy be investing in research seeking to better understand the use, production, and traffic of those drugs.^19^ However, studies on the prevalence of drug use among truck drivers, in Brazil, are not yet very numerous, only few of them analyze the factors related to drug use by those professionals,^6^ especially regarding amphetamine use.

Besides that, no Brazilian studies evaluated the interference of occupational factors (as provided by Law 12,619/2012, which is most commonly known as Resting Act, or “Lei do Descanso”, in Portuguese,^b^ which regulates the working hours and driving periods for professional drivers) on amphetamine use from truck drivers. No studies considered mental health factors to as variables which could influence amphetamine use either.^9^ ^,^ ^18^

Thus, this study aimed at testing whether occupational conditions from professional truck drivers are associated with amphetamine use, after demographic, mental health, and drug use characteristics are controlled for.

## METHODS

It is a cross-sectional study with a non-probabilistic sample with 684 truck drivers who were driving on three highways in Sao Paulo state (Presidente Dutra, Fernao Dias, and Cônego Domênico Rangoni), who had been recruited at *Serviço Social do Transporte* (SEST – Social Transportation Service) and *Serviço Nacional de Aprendizagem do Transporte *(SENAT – National Transportation Learning Service) stations, from June 2012 to September 2013. As the research which originated this study aimed to evaluate the effects from use of psychotropic substances on the level of attention of truck drivers, subjects who were found have at least one of the following health conditions – which could interfere in their evaluation – were excluded from the sample: subjects who had trouble seeing colors; subjects who were taking psychotropic medications; subjects who reported having brain trauma (SF) during in their lifetime; subjects who lost consciousness at least once in their lifetime; the ones with neurological disease histories; or drivers who were HIV-positive.

Truck drivers talked to a recruiter from the team in the related SEST-SENAT stations, and were informed the study would be conducted on that day and time. The study subjects were asked to answer a structured questionnaire, which had already been used by Leyton et al^11^ with Brazilian truck drivers, so demographic and occupational data could be collected.

Subjects were asked about their personal experiences with use of amphetamines and other drugs in their lifetime (defined as experimental use; that is, at least once in their lifetimes), in the twelve months prior (in that year; that is, at least once in the twelve months prior to the interview), and within the previous thirty days,^20^ regardless of the reasons why they used it. The criteria in the alcohol, smoking, and substance involvement screening test (ASSIST-WHO, 3.1 version)^7^ were also included in that questionnaire, to evaluate the risk for addiction to amphetamines and other drugs. The thirty points which were possible in the scale were classified as low risk (occasional use; up to three points), moderate risk (regular use, from three to 26 points), and high risk (problematic use, above 27 points).

Subjects also answered questionnaires about their mental health conditions, as those could be related to amphetamine use – they included the following: emotional stress (Lipp Stress Symptoms Inventory for Adults – LSSI), psychiatric disorders (Mini International Neuropsychiatric Interview Plus – MINI Plus), and sleep quality (Pittsburgh Sleep Quality Index – PSQI and Epworth Sleepiness Scale – ESE). Regarding their sleep quality, scores above five in the PSQI scale indicate bad sleep quality. In the ESE scale, scores above 10 indicate excessive sleepiness, and, above 16, high-level sleepiness.

All questionnaires and scales were double typed on Epi Info v.6.0 software. Consistency information checks and related corrections were performed. The data were transferred to and analyzed by R software, version 2.15.1. The classification variables were expressed as percentages and numerical ones (initially expressed as averages and standard deviations) were later classified according to their median values. Amphetamine use in the previous twelve months was the dependent variable, which allowed classifying subjects in users and non-users. The univariate analyses (two-tailed distribution) were conducted through chi-squared and Fisher’s tests. Independent variables “daily workload (hours)” and “restless driving (hours)” were classified according to the requirements from the Resting Act (Law 12,619/2012).^b^ Also, as high risk for amphetamine addiction was not very prevalent, that category was grouped with moderate risk as per ASSIST-WHO. Variables which reached p < 0.25 were included in a logistic regression model and selected through backward elimination. The odds ratio (OR and 95%CI) was calculated as a measure for association. The null hypothesis was rejected when p < 0.05. The model adjustment was evaluated by means of the analysis of standard deviation and Cook’s distance outliers. Multicollinearity was diagnosed by its impact on parameter estimates and by the Variance Inflation Factor (VIF).

This research was approved by the Research Ethics Committee from the Faculdade de Medicina of Universidade de São Paulo (Protocol 377/11, on 10/19/2011). All subjects signed consent forms (termo de consentimento livre e esclarecido) and were evaluated individually in close, safe, and silent rooms which had been specifically set aside for the research.

## RESULTS

Out of the 684 truck drivers who took part in the study, 149 (22.0%) were excluded for meeting at least one of the exclusion criteria of the original research. Also, twenty-one drivers were excluded as they reported having used illegal drugs other than amphetamines in that year. Thus, 514 drivers took part in the final analysis.

Subjects were also found to have an average age of 36.7 (SD = 7.8) years, average education level of 8.6 (SD = 2.3) years. Most of them (82.1%) were married or separated at the time of the interview. Subjects had an average of 12.7 (SD = 8.2) years of experience as professional drivers, driving an average 12.2 (SD = 3.9) hours daily, of which 4.7 (SD = 2.4) hours without resting, covering an average 1,159.7 (SD = 1,032.2) km on their last reported trip. More than half (59.9%) of subjects worked for companies, and 21.6% worked night or irregular shifts (Table 1).

**Table 1:** Demographic and occupational characteristics of truck drivers, according to their use of amphetamines in the 12 months prior to their interviews. Highways in Sao Paulo state, Southeastern Brazil, 2012-2013. (N = 514)

Variable	Amphetamine user								
	Total		Yes		No		OR	95%CI	p

	n	%	n	%	n	%			
Age (years)	36.7 (SD = 7.8)		38.1 (SD = 7.9)		33.4 (SD = 6.6)				
≤ 38	302	58.9	187	51.4	115	77.2	3.20	2.08;5.00	< 0.001*
> 38	211	41.1	177	48.6	34	21.8	1		
Schooling (years)	8.6 (SD = 2.3)		8.6 (SD = 2.3)		8.6 (SD = 2.2)				
≤ 9	305	59.4	210	57.5	95	63.8	1.29	0.88;1.92	0.193
> 9	209	40.6	155	42.5	54	35.2	1		
Marital status									
Single	94	17.9	56	15.3	38	25.5	1.88	1.19;3.00	< 0.001*
Non-single	420	82.1	309	84.7	111	74.5	1		
Experience length in the profession (years)	12.7 (SD = 8.2)		13.4 (SD = 8.6)		11.0 (SD = 6.7)				
≤ 11	268	52.2	178	48.8	90	60.4	1.60	1.09;2.36	0.017*
> 11	246	47.8	187	51.2	59	39.6	1		
Type of service									
Employed	307	59.9	227	62.4	80	53.7	1		0.069
Freelance	206	40.1	137	37.6	69	46.1	1.43	0.97;2.10	
Work shift									
Day	403	78.4	302	82.8	101	67.8	1		< 0.001*
Night or otherwise	111	21.6	63	17.2	48	32.2	2.28	1.47;3.53	
Daily working hours	12.2 (SD = 3.9)		11.7 (SD = 3.9)		13.3 (SD = 3.6)				
≤ 12	340	66.2	259	71.0	81	54.4	1		< 0.001*
> 12	174	33.8	106	29.0	68	45.6	2.05	1.38;3.04	
Restless working hours	4.7 (SD = 2.4)		4.6 (SD = 2.4)		5.1 (SD = 2.3)				< 0.001*
≤ 4	295	57.4	223	61.1	72	48.3	1		
> 4	219	42.6	142	39.9	77	51.7	1.68	1.14;2.46	
Distance traveled (km)	1159.7 (SD = 1032.2)		1014.5 (SD = 959.6)		1515.6 (SD = 1118.6)				< 0.001*
≤ 750	188	50.0	147	55.1	41	37.6	1		
> 750	188	50.0	120	44.9	68	62.4	2.03	1.29;3.21	
Total	514	100	364	71.0	149	29.0			

* Statistically significant values.

Regarding their mental health, 77.0% of subjects reported having used alcohol within the year prior to the interview, and 4.7%, at least one illegal drug (other than amphetamines) within the same period. Moreover, 56.0% of subjects scored for bad sleep quality, 33.7% of them experienced moderate to serious daytime sleepiness, 11.1% suffered from emotional stress, and 6.6% of drivers suffered from psychiatric disorders (Table 2).

**Table 2:** Use of alcohol, other drugs, and other mental health aspects from truck drivers, according to their use of amphetamines in the 12 months prior to their interviews. Highways in Sao Paulo state, Southeastern Brazil, 2012-2013. (N = 514)

Variable	Amphetamine user								
	Total		Yes		No		OR	95%CI	p

	n	%	n	%	n	%			
Use of psychoactive substances (year)									
Alcohol	396	77.0	270	74.0	126	84.6	1.92	1.17;3.18	< 0.010*
Other illegal drugs	24	4.7	1	0.3	23	15.4	66.44	8.88;497.6	< 0.001*
Mental health									
Sleep quality (PSQI)									
Good (< 5)	226	44.0	165	45.2	61	50.0	1		0.377
Bad (≤ 5)	288	56.0	200	54.8	88	50.0	1.19	0.81;1.75	
Excessive daytime sleepiness (EDS)									0.805
Normal (≤ 10)	341	66.3	242	66.3	99	66.4	1		
Moderate (10 < score ≤ 15)	137	26.7	99	27.1	38	25.5	0.93	0.60;1.46	
Serious (≥ 16)	36	7.0	24	6.6	12	8.1	1.22	0.59;2.54	
Number of hours of sleep	6.8 (SD = 1.7)		6.9 (SD = 1.6)		6.5 (SD = 1.9)				0.079
≤ 7	311	60.5	212	58.1	99	66.4	1.43	0.96;2.12	
> 7	203	39.5	153	41.9	50	33.6	1		
Stress (LSSI)	57	11.1	38	10.4	19	12.8	1.25	069;2.25	0.449
Psychiatric disorder (MINI Plus)	34	6.6	23	6.3	11	7.4	1.18	0.56;2.59	0.660
Total	514	100	364	71.0	149	29.0			

LSSI: Lipp Stress Symptoms Inventory for Adults; MINI Plus: Mini International Neuropsychiatric Interview Plus; PSQI: Pittsburgh Sleep Quality Index; ESS: Epworth Sleepiness Scale

* Statistically significant values.

Most of them (58.0%; n = 298) (95%CI 53.6;62.3) reported having used amphetamines in lifetime; 29.0% of them (n = 149) (95%CI 25.1;33.1) within a year; and 14.4% (n = 74) (95%CI 11.5;17.7), within a month. Among subjects who reported having used amphetamines within a year, 38.9% of them (n = 58) scored for problems related to such use as per the criteria from ASSIST-WHO.

The drivers who reported using amphetamines are often single and younger than non-users. Also, they are frequently less experienced as professional drivers, besides working more hours a day, driving more hours without resting – and for longer distances. They work night or irregular shifts more often as compared to non-users (Table 1). In regards to their mental health, use of alcohol was more frequent among amphetamine users. Amphetamine use was not associated with sleep conditions, emotional stress, or psychiatric disorders (Table 2).

After demographic and occupational variables were controlled for, the following from Table 3 were found: a) subjects who were younger than 38 years had higher chances of using amphetamines than their older peers; b) subjects with education levels below nine years had higher chances of using amphetamines than subjects with higher education levels; c) subjects who performed freelance work had higher chances of using amphetamines than employed peers; d) subjects who worked night or irregular shifts had higher chances for using amphetamines than their peers who only worked day shift; e) subjects who worked over 12 hours daily had increased chances for using amphetamines than their peers who worked up to 12 hours daily; e) drivers who stated having drunk alcohol at least once that year had increased chances for using amphetamines than their peers who reported not being users. Even though at a difference which is marginally significant, subjects who drove over four non-stop, no-resting hours had increased chances of using amphetamines than their peers who worked up to four hours.

**Table 3:** Adjusted and unadjusted models on factor related to amphetamine use among truck drivers as self-reported regarding the twelve months prior to their interviews. Highways in Sao Paulo state, Southeastern Brazil, 2012-2013. (N = 514)

Variable	No adjustment		p	After the adjustment*		p
	OR	95%CI		OR	95%CI	
Age (years)			< 0.001*			< 0.001*
≤ 38	3.20	2.08;5.00		3.69	2.32;6.01	
> 38	1			1		
Schooling (years)			0.193			0.011*
≤ 9	1.29	0.88;1.92		1.76	1.14;2.74	
> 9	1			1		
Marital status			< 0.001*			
Single	1.88	1.19;3.00*		–	–	–
Non-single	1			–	–	–
Years in the profession			< 0.05*			
≤ 11	1.60	1.09;2.36*		–	–	–
> 11	1			–	–	–
Type of service			0.069			0.020*
Employed	1			1		
Freelance	1.43	0.97;2.10		1.65	1.08;2.53	
Work shift			< 0.001*			0.003*
Daytime	1			1		
Night or otherwise	2.28	1.47;3.53*		2.05	1.27;3.29	
Daily working hours			< 0.001*			< 0.001*
≤ 12	1			1		
> 12	2.05	1.38;3.04		2.14	1.40;3.28	
Restless working hours			< 0.001*			
≤ 4	1			1		0.073
> 4	1.68	1.14;2.46		1.46	0.96;2.22	
Distance traveled (km)			< 0.001*			
≤ 750	1			–	–	–
> 750	2.03	1.29;3.21*		–	–	–
Alcohol use (12-month)			< 0.05*			
No	1			1		0.043*
Yes	1.92	1.17;3.18		1.74	1.03;3.03	
Other illegal drugs use (12-month)			< 0.001*			
No	1			–	–	–
Yes	66.44	8.88;497.6		–	–	–
Number of hours of sleep			0.079			
≤ 7	1.28	0.96;1.72		–	–	–
> 7	1			–	–	–

* The effect from mentioned variables as described in Table 3 was controlled for in the adjusted model, with the addition of variables quality of sleep (PSQI), excessive daytime sleepiness (EDS), stress, and existing psychiatric disorders.

## DISCUSSION

The results showed that bad occupational conditions for professional truck drivers interfere on amphetamine use, after the effect from demographic, mental health, and use of other drugs are controlled for. Although it is not possible to conjecture what the cause-effect relationship is in the association herein found, the study points towards the importance for responsible authorities to be alert to the organization of those professionals’ occupational activity, to prevent amphetamine use, its effects on driving, and other possible negative outcomes in traffic.

The subjects in this study were young, low schooled adults who worked long hours daily, corroborating previous studies.^9^ ^,^ ^18^

Almost one third of subjects (29.0%) reported having used amphetamines in that year, which is similar to the 29.5% found by Girotto et al.^6^

Thus, no person can deny truck drivers keep using amphetamines even after the introduction of RDC (Resolution from the Collegiate Board of Directors) 52/2011 by ANVISA. The similar results from this study and the one by Girotto et al^6^ may suggest that prevalences for amphetamine use have not changed after RDC 52/2011 was implemented. However, one cannot dismiss the highly variable rates for amphetamine use in Brazil,^4^ ^,^ ^9^ ^,^ ^18^ which may both justify a certain regionalization in its use and its being an artifact of methodological differences between surveyed studies, such as the employment of different evaluation measurements (use in lifetime, in a year, in a month).

On the other hand, prevalence of amphetamine use among the subjects in this study was much higher than 1.1% – which is the index that was identified for the Brazilian population at large –,^c^ which suggests its use keeps being part of culture among truck drivers.

In turn, the results from the logistic regression model pointed the following variables as predictors for amphetamine use: being younger than 38 years, having attended school for nine years or less, being freelancers, working night or irregular shifts, working over 12 hours daily, and drinking alcohol.

In fact, the association between amphetamine use and young truck drivers corroborates the findings from Knauth et al.^9^ Thus, the relationship between amphetamine use and age may be possibly mediated by these young adults’ low experience as professional drivers. In this sense, Williamson^22^ suggested that less experienced professional drivers are more prone to using stimulants, possibly because they do not know how to deal with the difficulties in their profession, thus resorting to drugs as a possible solution.

In turn, the association between amphetamine use and drivers’ low education may suggest that those professionals are not aware of the future outcomes of such use, especially regarding their possibity to develop amphetamine-related disorders such as abuse or dependence. Thus, the finding that 38.9% of subjects stated they took amphetamines and suffered from the possible problems related to such use indicates that they could actually already be the target for specialized intervention strategies. Because the undesired effects from amphetamine use are commonly unknown to truck drivers,^12^ this finding reinforces the need for investments in public policies intending on educating that social segment.

Drinking alcohol was also highlighted among amphetamine use predictors, which then again confirms the previous results by Knauth et al.^9^ In this sense, drinking alcohol was more prevalent among truck drivers (77.0%) than among men in the general Brazilian population (use in that year: 62.0%).^c^ Also, use of alcohol among truck drivers exceeded the estimated levels for the general Brazilian population.^21^ ^,^ ^c^ Those results suggest that those professionals may engage in polydrug use, a problematic use pattern, which leads to serious consequences to users and to public health and safety.^20^

In regards to the occupational variables, working over 12 hours daily kept being a predictor for amphetamine use. Corroborating that result, the association between excess workloads and amphetamine use had been previously pointed out.^9^ ^,^ ^18^ Knauth et al^9^ found that trips lasting over three days increased the risk of amphetamine use among truck drivers, whereas Sinagawa et al^18^ found truck drivers who covered over 270 km to have more chances of using amphetamines than their peers who traveled smaller distances. However, those authors have not controlled for mental health effects on amphetamine use, nor have they contextualized their findings with the presidential decree which regulates amphetamine production, or even with the legislation governing the activities from professional long-haul drivers. However, in conclusion, all those data corroborate the use of amphetamines by truck drivers intending on increasing awareness, due to the need of traveling longer distances, as previously described.^9^ ^,^ ^11^

Also, driving for over four hours non-stop also kept being a predictor for amphetamine use, with a marginally significant difference, however (p = 0.073). In addition to the aforementioned finding, those information point towards the need for complying with the provisions of Law 12,619/2012, the so-called Resting Act (*Lei do Descanso*).^b^ That law ensures drivers a minimum one-hour meal break, as well as a daily 11-hour resting period, which suggests a working routine of up to 12 hours. Hence, professionals who drive for over 12 hours daily, besides failing to comply with the law, are more prone to using amphetamines. That information corroborates the findings from Williamson,^22^ who showed that truck drivers who make use of stimulants violate traffic laws, especially those related to working hours. The danger in that violation is observed through the fact that drivers who work over 12 hours a day are more prone to fatigue and its outcomes.^2^ Thus, van der Beek^2^ proposed that reducing the number of working hours be one of the strategies for protecting the health and safety of truck drivers, which increases their chances of properly recovering after a typical day of work, and additionally reducing their chances of getting involved in traffic accidents.

Being a freelance or working night or irregular shifts increased the risk of amphetamine use. In fact, as stated by Moreno et al,^12^ freelance drivers are more dependent on work demands, thus commonly engaging in long-haul trips – which involve irregular work shifts. And drivers who work irregular shifts are the ones who most frequently take stimulants.^16^ Thus, this is evidence in favor of inspecting daily work schedules of truck drivers in Brazil. Law 12,619/2012^b^ presently only regulates the activity of professional drivers who are employed – those who are freelance are not inspected.

Use of amphetamines among truck drivers increases the chance of risky behaviors in traffic by 78.0%, which can be fatal.^5^ Brodie et al^3^ found that one in every six truck drivers who died in traffic accidents had traces of amphetamines in their blood samples. That way, amphetamine use among professional drivers is a very important risk factor which can be associated with serious accidents that sometimes lead to their deaths, as well as irreversible outcomes to third parties, which negatively impact society and public budgets.

In that scenario, the approval of Legislative Decree 273/2014 is concerning,^d^ as it canceled Resolution 52/2011 from ANVISA,^a^ which prohibited amphetamine production and use. Although there is still a long way until those substances are effectively released in the market, that will make it easier for truck drivers to purchase them, consequently assuming all associated risks. Between 2011 and 2014, informal reports already indicated those professionals had already been adopting other stimulates (such as cocaine) as amphetamine replacements, in a way that resorting to them again could add comorbidities to the psychiatric profiles of those professionals.

Besides focusing on controlling the sale of amphetamines, public authorities must enforce the law which regulates the organization of truck drivers’ professional activities and create proper conditions for Law 12,619/2012 to be complied with.^b^ In this sense, a higher number of safe places may be established, to provide truck drivers with spaces in which they can pull over and rest, so they have conditions to perform dignified work, as recommended by the International Labor Organization.^e^ Also, due to their polydrug use, policies to fight alcohol use may have effects on use of amphetamines and vice-versa.

In conclusion, the early recognition of drivers who make use of amphetamines and other drugs must be fostered. Educational campaigns are also required to be developed, to promote knowledge that is specific to that population. Drivers who are already experiencing problems due to amphetamine use should be sent to specialized programs, to get medicated, be provided psychological counseling, and rehabilitation, aiming to have decreased chances of relapsing and improved safety in traffic.^1^

This study only reflects a specific aspect of the situation. The use of a non-probabilistic sample is another limiting factor, as the data cannot be assumed to reflect the whole population of truck drivers in Brazil.

## Footnotes

a Ministério da Saúde. Agência Nacional de Vigilância Sanitária. Resolução - RDC nº 52, de 6 de outubro de 2011. Dispõe sobre a proibição do uso das substâncias anfepramona, femproporex e mazindol, seus sais e isômeros, bem como intermediários e medidas de controle da prescrição e dispensação de medicamentos que contenham a substância sibutramina, seus sais e isômeros, bem como intermediários e dá outras providências. *Diário Oficial União*. 10 out 2011;seção 1:55 [cited 2014 Oct 15]. Available from: http://bvsms.saude.gov.br/bvs/saudelegis/anvisa/2011/res0052_06_10_2011.html

b Brasil. Lei nº 12.619, de 30 de abril de 2012. Dispõe sobre o exercício da profissão de motorista; altera a Consolidação das Leis do Trabalho - CLT, aprovada pelo Decreto-Lei nº 5.452, de 1º de maio de 1943, e as Leis nos 9.503, de 23 de setembro de 1997, 10.233, de 5 de junho de 2001, 11.079, de 30 de dezembro de 2004, e 12.023, de 27 de agosto de 2009, para regular e disciplinar a jornada de trabalho e o tempo de direção do motorista profissional; e dá outras providências. *Diário Oficial União*. 5 maio 2012;seção 1:5 [cited 2014 Oct 15]. Available from: http://www.planalto.gov.br/ccivil_03/_ato2011-2014/2012/lei/l12619.htm

c Universidade Federal de São Paulo, Instituto Nacional de Ciência e Tecnologia para Políticas Públicas de Álcool e Outras Drogas (INPAD). Segundo levantamento nacional de álcool e drogas: relatório 2012. São Paulo (SP): Universidade Federal de São Paulo; 2014.

d Brasil. Decreto Legislativo nº 273, de 2014. Susta a Resolução - RDC nº 52, de 6 de outubro de 2011, da Agência Nacional de Vigilância Sanitária - ANVISA, que dispõe sobre a proibição do uso das substâncias anfepramona, femproporex e mazindol, seus sais e isômeros, bem como intermediários e medidas de controle da prescrição e dispensação de medicamentos que contenham a substância sibutramina, seus sais e isômeros, bem como intermediários. *Diário Oficial União*. 5 set 2014;seção 1:1.

e International Labor Organization (ILO). Decent worg agenda. Geneve: International Labor Organization; 2013 [cited 2014 Oct 15]. Available from: http://www.ilo.org/global/about-the-ilo/decent-work-agenda/lang--en/index.htm
