# Supplementary material for: Occupational conditions and the risk of the use of amphetamines by truck drivers
Source: Rev Saude Publica. 2015 Sep 29;49:61. doi: 10.1590/S0034-8910.2015049005944 (PMC4617435; doi:10.1590/S0034-8910.2015049005944)
Supplement: Translation

# Translation: Condições ocupacionais e o risco de uso de anfetaminas entre motoristas de caminhão

**Authors:** Lúcio Garcia de Oliveira, Letícia Maria de Araújo de Souza, Lúcia Pereira Barroso, Marcela Júlio César Gouvêa, Carlos Vinícius Dias de Almeida, Daniel Romero Muñoz, Vilma Leyton

**Affiliations:** I Departamento de Medicina Legal, Ética Médica, Medicina Social e do Trabalho. Faculdade de Medicina. Universidade de São Paulo. São Paulo, SP, Brasil; II Departamento de Estatística. Instituto de Matemática e Estatística. Universidade de São Paulo. São Paulo, SP, Brasil

**Keywords:** Transportes, recursos humanos, Anfetaminas, Condições de Trabalho, Fatores de Risco, Riscos Ocupacionais, Saúde do Trabalhador, Estudos Transversais

## Abstract

**OBJETIVO:**

Testar se as condições ocupacionais de motoristas profissionais de caminhão estariam associadas ao uso de anfetaminas após o controle do efeito de características demográficas, de saúde mental e uso de drogas.

**MÉTODOS:**

Estudo transversal, com amostra não-probabilística de 684 motoristas de caminhão, do sexo masculino, recrutada em três rodovias do estado de São Paulo, durante os anos de 2012 e 2013. Foram coletadas informações demográficas, ocupacionais, sobre uso de drogas e sobre saúde mental (qualidade de sono, estresse emocional e transtornos psiquiátricos). Foi desenvolvido um modelo de regressão logística para identificar os fatores associados ao uso de anfetaminas. A razão de chances (OR; IC95%) foi definida como a medida de associação e o nível de significância considerado foi p < 0,05.

**RESULTADOS:**

A amostra estudada apresentou idade média de 36,7 (DP = 7,8) anos e baixa escolaridade (8,6 [DP = 2,3] anos). O uso de anfetaminas nos doze meses prévios à entrevista foi autorrelatado por 29,0% dos motoristas. Após o controle do efeito de variáveis demográficas e ocupacionais, os fatores preditores do uso de anfetaminas entre os motoristas de caminhão foram: ter menos de 38 anos (OR = 3,69), menos de nove anos de escolaridade (OR = 1,76), ser autônomo (OR = 1,65), trabalhar em turno noturno ou turno irregular (OR = 2,05), trabalhar mais de 12 horas diárias (OR = 2,14) e usar álcool (OR = 1,74).

**CONCLUSÕES:**

Os aspectos ocupacionais estão intimamente associados ao uso de anfetaminas entre motoristas de caminhão, reforçando a importância da fiscalização da lei (Lei do Descanso; Lei 12.619/2012) que regulamenta a carga horária de trabalho desses profissionais. Os resultados mostram a necessidade de haver maior rigor sobre a comercialização e prescrição das anfetaminas no Brasil.

## INTRODUÇÃO

Em 6 de outubro de 2011, a Agência Nacional de Vigilância Sanitária (Anvisa), pela Resolução da Diretoria Colegiada de número 52 (RDC 52/2011), proibiu a produção, comercialização, prescrição e, consequentemente, o consumo de anfepramona, femproporex e mazindol.^a^ Essas substâncias são conhecidas como derivados anfetamínicos, anfetaminas, estimulantes e anorexígenos, sendo essas duas últimas denominações devidas aos seus efeitos sobre o sistema nervoso central. A despeito disso, tais substâncias continuam sendo consumidas por motoristas de caminhão e, possivelmente, com a mesma magnitude de antes da citada regulamentação.^14^

Nesse contexto, condições laborais precárias têm encorajado os motoristas de caminhão a usarem anfetaminas,^4^ ^-^ ^6^ especialmente no cenário atual em que a indústria do transporte rodoviário tem se tornado cada vez mais competitiva.^2^ ^,^ ^22^ Dessa forma, as anfetaminas vêm sendo usadas dentro de um estilo de vida moderno para reduzir a fadiga e manter o foco e a vigília quando em situação laboral.^6^ ^,^ ^9^ ^,^ ^15^

Revisão sistemática^6^ mostrou que as anfetaminas são a segunda classe de drogas mais usadas entre motoristas de caminhão (perdendo apenas para as bebidas alcoólicas). Esse estudo mostrou que 29,5% dos motoristas relataram usar anfetaminas, enquanto 8,5% tinham algum vestígio dessas substâncias em amostras biológicas quando submetidas a procedimentos de análise toxicológica.^6^

Consistente com os motivos de uso já citados pelos motoristas de caminhão, as anfetaminas podem melhorar o funcionamento de atenção.^15^ Entretanto, o aumento da concentração plasmática dessas substâncias piora o desempenho na direção,^13^ por conta de euforia, fluxo rápido de ideias, aumento da sensação de habilidade mental e física e, até mesmo, pensamentos paranoides, alucinações e confusão mental.^23^ Em conjunto, esses efeitos prejudicam a coordenação motora, a concentração e o julgamento, qualidades necessárias durante a direção de qualquer veículo.^23^ Dessa forma, o uso das anfetaminas, entre outras substâncias psicotrópicas, não é condizente com a prática de uma direção segura.^17^ ^,^ ^23^

Nesse cenário, as anfetaminas continuam sendo as substâncias mais frequentemente encontradas em amostras biológicas de motoristas parados por mau desempenho na direção.^8^ Também estão presentes em amostras biológicas de motoristas fatalmente feridos^3^ ^,^ ^5^ ^,^ ^10^ e seu uso está associado a acidentes de trânsito,^4^ colocando em risco a segurança e saúde desses profissionais e o bem-estar da população.^19^

Como resposta ao problema do uso das anfetaminas, a Organização das Nações Unidas (ONU) sugere que a estratégia mais oportuna e precisa de controle seja o investimento em pesquisas para melhor compreensão sobre seu uso, produção e tráfico.^19^ Entretanto, estudos sobre a prevalência do uso de drogas entre motoristas de caminhão, no Brasil, ainda são incipientes e, sobretudo, poucos deles são analíticos e objetivam conhecer os fatores relacionados ao uso de drogas entre esses profissionais,^6^ particularmente em relação ao uso das anfetaminas.

Além disso, nenhum estudo nacional avaliou a interferência de fatores ocupacionais (conforme o regulamentado pela Lei 12.619/2012, popularmente conhecida como Lei do Descanso,^b^ que disciplina a jornada de trabalho e o tempo de direção do motorista profissional) sobre o uso das anfetaminas entre motoristas de caminhão. E também nenhum incluiu fatores de saúde mental às possíveis variáveis explicativas do uso de anfetaminas.^9^ ^,^ ^18^

Assim, o presente estudo teve como objetivo testar se as condições ocupacionais de motoristas profissionais de caminhão estariam associadas ao uso de anfetaminas após o controle do efeito de características demográficas, de saúde mental e uso de drogas.

## MÉTODOS

Estudo transversal com amostra não-probabilística de 684 motoristas de caminhão que circulavam por três rodovias do Estado de São Paulo (Presidente Dutra, Fernão Dias e Cônego Domênico Rangoni), recrutados em postos de atendimento das entidades civis Serviço Social do Transporte (SEST) e Serviço Nacional de Aprendizagem do Transporte (SENAT), de junho de 2012 a setembro de 2013. Como a pesquisa que deu origem a este estudo visou avaliar os efeitos do uso de substâncias psicotrópicas sobre o funcionamento de atenção de motoristas de caminhão, os sujeitos que apresentassem pelo menos uma condição de saúde, que interferisse nessa avaliação, foram excluídos da amostra: ter dificuldade para a visualização de cores; estar sob o uso terapêutico de medicamentos psicoativos; relatar o acontecimento de traumatismo crânio-encefálico (TCE) na vida; ter sofrido algum episódio com perda de consciência na vida; ter histórico de doenças neurológicas; ou ser soroposito para HIV.

Os motoristas de caminhão foram abordados por um recrutador da equipe, nos citados postos da SEST-SENAT, e informados sobre a realização da pesquisa naquele dia e horário. Os participantes da pesquisa foram solicitados a responder um questionário estruturado, já utilizado por Leyton et al^11^ com motoristas de caminhão brasileiros, para o registro de informações demográficas e ocupacionais.

Aos participantes foi questionada a experiência pessoal com o uso de anfetaminas e outras drogas na vida (definido como o uso experimental, ou seja, pelo menos uma vez na vida), nos últimos doze meses (no ano, ou seja, pelo menos uma vez nos doze meses que antecederam a entrevista) e nos últimos trinta dias,^20^ independentemente da razão de uso. Os critérios do teste de triagem do envolvimento com álcool, cigarro e outras substâncias (ASSIST-WHO, versão 3.1),^7^ foram incluídos nesse questionário a fim de avaliar o risco de desenvolvimento de dependência, associado ao uso de anfetaminas e outras drogas. Os trinta pontos possíveis na escala foram categorizados em risco baixo (uso ocasional; até três pontos), risco moderado (uso regular; de três a 26 pontos) e risco alto (uso problemático; acima de 27 pontos).

Os participantes também responderam escalas para a avaliação de condições de saúde mental que pudessem ter relação com o uso de anfetaminas, como: estresse emocional (Inventário de Sintomas de Estresse para Adultos de Lipp – ISSL), transtornos psiquiátricos (*Mini International Neuropsychiatric Interview Plus* – MINI Plus) e qualidade de sono (Índice de Qualidade de Sono de Pittsburgh – PSQI e Escala de Sonolência de Epworth – ESE). Em relação à qualidade de sono, pontuações acima de cinco na escala PSQI indicam qualidade de sono ruim. Na escala ESE, pontuações acima de 10 indicam sonolência excessiva e acima de 16, sonolência de alto nível.

Todos os questionários e escalas foram submetidos à digitação dupla no *software* Epi Info v.6.0. Checagens de consistência da informação e correções pertinentes foram realizadas. Os dados foram transferidos e analisados no programa R, versão 2.15.1. As variáveis categóricas foram expressas em porcentagens e as numéricas (inicialmente expressas em média e desvio padrão) foram posteriormente categorizadas conforme o valor da mediana. A variável-desfecho foi o uso de anfetaminas no ano, possibilitando a categorização dos participantes em usuários e não-usuários. As análises univariadas (distribuição bicaudal) foram realizadas pelos testes Qui-quadrado e Fisher. As variáveis explicativas “carga diária de trabalho” (horas) e “direção sem descanso” (horas) foram categorizadas conforme os pressupostos da Lei do Descanso (Lei 12.619/2012).^b^ Ainda, como o risco alto para o desenvolvimento de dependência às anfetaminas foi pouco prevalente, essa categoria foi agrupada ao risco moderado do ASSIST-WHO. As variáveis que atingiram p < 0,25 foram incluídas em um modelo de regressão logística e selecionadas pelo método de *backward elimination*. A razão de chances (OR e IC95%) foi calculada como medida de associação. A hipótese nula foi refutada quando p < 0,05. O ajuste do modelo foi avaliado pela análise do resíduo componente do desvio e da distância de Cook. O diagnóstico de multicolinearidade foi avaliado pelo impacto nas estimativas dos parâmetros e por meio do *Variance Inflation Factor* (VIF).

A pesquisa que deu origem a este estudo foi aprovada pelo Comitê de Ética em Pesquisa da Faculdade de Medicina da Universidade de São Paulo (Protocolo 377/11, em 19/10/2011)). Todos os participantes assinaram termo de consentimento livre e esclarecido e foram avaliados individualmente, em ambientes fechados, seguros e silenciosos, destinados exclusivamente à realização desta pesquisa.

## RESULTADOS

Dos 684 motoristas de caminhão que participaram do estudo, 149 (22,0%) foram excluídos por terem preenchido pelo menos um dos critérios de exclusão da pesquisa original. Ainda, vinte e um motoristas foram excluídos por terem relatado o uso de outra droga ilícita, à exceção das anfetaminas, no ano. Assim, 514 motoristas participaram da análise final.

Os participantes apresentaram média de idade de 36,7 (DP = 7,8) anos, média de escolaridade de 8,6 (DP = 2,3) anos e a maioria (82,1%) estava casada ou separada no momento da entrevista. Os participantes tinham em média 12,7 (DP = 8,2) anos de experiência como motoristas profissionais e dirigiam média diária de 12,2 (DP = 3,9) horas, das quais 4,7 (DP = 2,4) horas sem descanso, percorrendo, em média, 1.159,7 (DP = 1.032,2) km na última viagem referida. Mais da metade (59,9%) dos participantes estava contratada por empresas e 21,6% trabalhavam em turno noturno ou irregular (Tabela 1).

**Tabela 1:** Características demográficas e ocupacionais de motoristas de caminhão, segundo uso de anfetaminas nos 12 meses prévios à entrevista. Rodovias do Estado de São Paulo, Brasil, 2012-2013. (N = 514)

Variável	Usuário de anfetaminas								
	Total		Sim		Não		OR	IC95%	p

	n	%	n	%	n	%			
Idade (anos)	36,7 (DP = 7,8)		38,1 (DP = 7,9)		33,4 (DP = 6,6)				
≤ 38	302	58,9	187	51,4	115	77,2	3,20	2,08;5,00	< 0,001*
> 38	211	41,1	177	48,6	34	21,8	1		
Escolaridade (anos)	8,6 (DP = 2,3)		8,6 (DP = 2,3)		8,6 (DP = 2,2)				
≤ 9	305	59,4	210	57,5	95	63,8	1,29	0,88;1,92	0,193
> 9	209	40,6	155	42,5	54	35,2	1		
Estado civil									
Solteiro	94	17,9	56	15,3	38	25,5	1,88	1,19;3,00	< 0,001*
Não-solteiro	420	82,1	309	84,7	111	74,5	1		
Experiência na profissão (anos)	12,7 (DP = 8,2)		13,4 (DP = 8,6)		11,0 (DP = 6,7)				
≤ 11	268	52,2	178	48,8	90	60,4	1,60	1,09;2,36	0,017*
> 11	246	47,8	187	51,2	59	39,6	1		
Tipo de serviço									
Contratado	307	59,9	227	62,4	80	53,7	1		0,069
Autônomo	206	40,1	137	37,6	69	46,1	1,43	0,97;2,10	
Turno de trabalho									
Dia	403	78,4	302	82,8	101	67,8	1		< 0,001*
Noturno e outros	111	21,6	63	17,2	48	32,2	2,28	1,47;3,53	
Jornada diária (horas)	12,2 (DP = 3,9)		11,7 (DP = 3,9)		13,3 (DP = 3,6)				
≤ 12	340	66,2	259	71,0	81	54,4	1		< 0,001*
> 12	174	33,8	106	29,0	68	45,6	2,05	1,38;3,04	
Jornada sem descanso (horas)	4,7 (DP = 2,4)		4,6 (DP = 2,4)		5,1 (DP = 2,3)				< 0,001*
≤ 4	295	57,4	223	61,1	72	48,3	1		
> 4	219	42,6	142	39,9	77	51,7	1,68	1,14;2,46	
Trajetória percorrida (km)	1159,7 (DP = 1032,2)		1014,5 (DP = 959,6)		1515,6 (DP = 1118,6)				< 0,001*
≤ 750	188	50,0	147	55,1	41	37,6	1		
> 750	188	50,0	120	44,9	68	62,4	2,03	1,29;3,21	
Total	514	100	364	71,0	149	29,0			

* Valores com significância estatística.

Em relação à saúde mental, 77,0% dos participantes referiram usar álcool no ano prévio à entrevista e 4,7% pelo menos outra droga ilícita (além das anfetaminas) no mesmo período. Ainda, 56,0% dos participantes tinham má qualidade de sono, 33,7% tinham sonolência diurna, de moderada a grave, 11,1% tinham estresse emocional e 6,6%, algum transtorno psiquiátrico (Tabela 2).

**Tabela 2:** Uso de álcool, de outras drogas e outros aspectos de saúde mental de motoristas de caminhão, segundo uso de anfetaminas nos 12 meses prévios à entrevista. Rodovias do Estado de São Paulo, Brasil, 2012-2013. (N = 514)

Variável	Usuário de anfetaminas								
	Total		Sim		Não		OR	IC95%	p

	n	%	n	%	n	%			
Uso de substâncias psicoativas (ano)									
Álcool	396	77,0	270	74,0	126	84,6	1,92	1,17;3,18	< 0,010*
Outras drogas ilícitas	24	4,7	1	0,3	23	15,4	66,44	8,88;497,6	< 0,001*
Saúde mental									
Qualidade de sono (PSQI)									
Boa (< 5)	226	44,0	165	45,2	61	50,0	1		0,377
Ruim (≤ 5)	288	56,0	200	54,8	88	50,0	1,19	0,81;1,75	
Sonolência excessiva diurna (ESE)									0,805
Normal (≤ 10)	341	66,3	242	66,3	99	66,4	1		
Moderada (10 < score < 15)	137	26,7	99	27,1	38	25,5	0,93	0,60;1,46	
Grave (≥ 15)	36	7,0	24	6,6	12	8,1	1,22	0,59;2,54	
Número de horas de sono	6,8 (DP = 1,7)		6,9 (DP = 1,6)		6,5 (DP = 1,9)				0,079
≤ 7	311	60,5	212	58,1	99	66,4	1,43	0,96;2,12	
> 7	203	39,5	153	41,9	50	33,6	1		
Estresse (ISSL)	57	11,1	38	10,4	19	12,8	1,25	069;2,25	0,449
Transtorno psiquiátrico (MINI Plus)	34	6,6	23	6,3	11	7,4	1,18	0,56;2,59	0,660
Total	514	100	364	71,0	149	29,0			

ISSL: Inventário de Sintomas de Estresse para Adultos de Lipp; MINI Plus: Mini International Neuropsychiatric Interview Plus; PSQI: Índice de Qualidade de Sono de Pittsburgh; ESE: Escala de Sonolência de Epworth

* Valores com significância estatística.

A maioria (58,0%; n = 298) (IC95% 53,6;62,3) relatou ter feito uso de anfetaminas na vida; 29,0% (n = 149) (IC95% 25,1;33,1) no ano; e 14,4% (n = 74) (IC95% 11,5;17,7), no mês. Entre os participantes que relataram ter usado anfetaminas no ano, 38,9% (n = 58) já estariam desenvolvendo algum problema relacionado a esse uso conforme os critérios do ASSIST-WHO.

Os motoristas que relataram usar anfetaminas são, frequentemente, solteiros e mais jovens que os não usuários de anfetaminas. Ainda, têm frequentemente menos experiência como motoristas profissionais, trabalham mais horas diárias, dirigem mais horas sem descanso, por maiores distâncias e mais frequentemente em turno noturno ou irregular quando comparados aos motoristas não usuários (Tabela 1). Quanto à saúde mental, o uso de álcool foi mais frequente entre os usuários de anfetaminas. Não houve associação entre o uso de anfetaminas e a condição de sono, estresse emocional e transtornos psiquiátricos (Tabela 2).

Após controle do efeito de variáveis demográficas e ocupacionais, foram observados os resultados constantes da Tabela 3: a) participantes com menos de 38 anos tiveram maiores chances de usar anfetaminas que os pares mais velhos; b) participantes com menos de nove anos de escolaridade tiveram maiores chances de usar anfetaminas que os pares de maior escolaridade; c) participantes que desempenhavam atividades de forma autônoma tiveram maiores chances de usar anfetaminas que os pares contratados; d) participantes que trabalhavam em turno noturno ou irregular tiveram maiores chances de usar anfetaminas que os pares que trabalhavam apenas em turno diurno; e) participantes que trabalhavam mais de 12 horas diárias tiveram mais chances de usar anfetaminas que os pares que trabalhavam até 12 horas diárias; e f) motoristas que declararam ter usado álcool no ano tiveram mais chances de usar anfetaminas que os pares que não relataram engajar nesse uso. Embora com diferença marginalmente significante, os participantes que dirigiam mais de quatro horas ininterruptas sem descanso tiveram mais chances de usar anfetaminas que os pares que trabalhavam até quatro horas.

**Tabela 3:** Modelo sem ajuste e ajustado sobre os fatores relacionados ao uso de anfetaminas entre motoristas de caminhão conforme o relato desse uso nos doze meses prévios à entrevista. Rodovias do Estado de São Paulo, Brasil, 2012-2013. (N = 514)

Variável	Sem ajuste		p	Após ajuste*		p
	OR	IC95%		OR	IC95%	
Idade (anos)			< 0,001*			< 0,001*
≤ 38	3,20	2,08;5,00		3,69	2,32;6,01	
> 38	1			1		
Escolaridade (anos)			0,193			0,011*
≤ 9	1,29	0,88;1,92		1,76	1,14;2,74	
> 9	1			1		
Estado civil			< 0,001*			
Solteiro	1,88	1,19;3,00*		–	–	–
Não-solteiro	1			–	–	–
Anos profissão			< 0,05*			
≤ 11	1,60	1,09;2,36*		–	–	–
> 11	1			–	–	–
Tipo de serviço			0,069			0,020*
Contratado	1			1		
Autônomo	1,43	0,97;2,10		1,65	1,08;2,53	
Turno de trabalho			< 0,001*			0,003*
Dia	1			1		
Noturno e outros	2,28	1,47;3,53*		2,05	1,27;3,29	
Jornada diária (horas)			< 0,001*			< 0,001*
≤ 12	1			1		
> 12	2,05	1,38;3,04		2,14	1,40;3,28	
Jornada sem descanso (horas)			< 0,001*			
≤ 4	1			1		0,073
> 4	1,68	1,14;2,46		1,46	0,96;2,22	
Trajetória percorrida			< 0,001*			
≤ 750	1			–	–	–
> 750	2,03	1,29;3,21*		–	–	–
Álcool			< 0,05*			
Não	1			1		0,043*
Sim	1,92	1,17;3,18		1,74	1,03;3,03	
Outras drogas ilícitas			< 0,001*			
Não	1			–	–	–
Sim	66,44	8,88;497,6		–	–	–
Número de horas de sono			0,079			
≤ 7	1,28	0,96;1,72		–	–	–
> 7	1			–	–	–

* No modelo ajustado foi controlado o efeito das variáveis citadas e descritas na Tabela 3, acrescentado das variáveis de qualidade de sono (PSQI), sonolência excessiva diurna (ESE), estresse e existência de algum transtorno psiquiátrico.

## DISCUSSÃO

Os resultados mostraram que más condições ocupacionais de motoristas profissionais de caminhão interferem sobre o uso de anfetaminas, após o controle do efeito de características demográficas, de saúde mental e do uso de outras drogas. Embora não seja possível especular sobre a relação causal da associação aqui encontrada, o estudo aponta para a importância de as autoridades competentes atentarem para a organização da atividade ocupacional desses profissionais e, sobretudo, a respeito da fiscalização das leis que a disciplinam, de forma a prevenir o uso de anfetaminas, seus efeitos sobre a direção e possíveis desdobramentos negativos no trânsito.

Os participantes desse estudo eram adultos jovens, de baixa escolaridade, que assumiam longas cargas diárias de trabalho, corroborando estudos prévios.^9^ ^,^ ^18^

Quase um terço dos participantes (29,0%) relatou ter feito o uso de anfetaminas no ano, semelhante à prevalência de 29,5% identificada por Girotto et al.^6^

Assim, é inegável a continuidade do uso de anfetaminas entre os motoristas de caminhão mesmo após a introdução da RDC 52/2011 pela Anvisa. A proximidade do resultado deste estudo de Girotto et al^6^ pode sugerir que a prevalência do uso de anfetaminas não mudou após a implementação da RDC 52/2011. Entretanto, também não se pode desconsiderar a alta variabilidade do uso de anfetaminas no País,^4^ ^,^ ^9^ ^,^ ^18^ o que pode justificar tanto uma regionalidade de uso quanto um artefato das diferenças metodológicas entre os estudos levantados, a exemplo do emprego de diferentes medidas de avaliação (uso na vida, no ano, no mês).

Por outro lado, a prevalência de uso de anfetaminas entre os participantes deste estudo foi muito superior aos 1,1% identificados para a população geral brasileira,^c^ sugerindo que esse uso continue sendo particular à cultura dos motoristas de caminhão.

Já os resultados do modelo de regressão logística apontaram as seguintes variáveis como preditores do uso de anfetaminas: ter idade inferior a 38 anos, escolaridade inferior a nove anos, ser autônomo, trabalhar em turno noturno ou irregular, trabalhar mais de 12 horas diárias e usar álcool.

De fato, a associação do uso de anfetaminas a motoristas de caminhão jovens é consistente com o estudo de Knauth et al.^9^ Assim, é possível que a relação entre o uso de anfetaminas e idade seja mediada pela pouca experiência desses jovens como motoristas profissionais. Nesse sentido, Williamson^22^ sugeriu que motoristas profissionais menos experientes estariam mais propensos ao uso de estimulantes, possivelmente por não saberem lidar com as diversidades da profissão, recorrendo, então, ao uso de drogas como uma possível solução.

Já a associação do uso de anfetaminas à baixa escolaridade do motorista pode sugerir o desconhecimento desses profissionais quanto aos desdobramentos futuros de tal uso, em particular, sobre a possibilidade de sua evolução a um estado de abuso ou dependência. Assim, a identificação de que 38,9% dos participantes, que haviam declarado usar anfetaminas, já estariam sofrendo problemas relacionados a esse uso, indica que, na verdade, já poderiam ser alvo de estratégias especializadas de intervenção. Como os efeitos indesejados do uso de anfetaminas são comumente desconhecidos pelos motoristas de caminhão,^12^ esse achado reforça a necessidade de investimentos em políticas públicas para a educação desse segmento social.

O uso de álcool também se destacou entre os preditores do uso de anfetaminas, corroborando novamente os resultados prévios de Knauth et al.^9^ Nesse sentido, o uso de álcool foi mais prevalente entre os motoristas de caminhão (77,0%) que entre os homens da população geral brasileira (uso no ano: 62,0%).^c^ Ainda, o uso de álcool entre os motoristas de caminhão ultrapassou os níveis avaliados para a população geral brasileira, especificamente entre os usuários de anfetaminas.^21^ ^,^ ^c^ Esses resultados sugerem que tais profissionais estejam engajados no uso múltiplo de substâncias, um padrão problemático de uso, que causa sérias consequências ao usuário e à saúde e segurança pública.^20^

Quanto às variáveis ocupacionais, trabalhar mais de 12 horas diárias permaneceu como preditor do uso de anfetaminas. Consistente com esse resultado, a associação entre o excesso de carga diária de trabalho e o uso de anfetaminas foi previamente apontada.^9^ ^,^ ^18^ Knauth et al^9^ apontaram que viagens de duração superior a três dias aumentavam o risco de uso de anfetaminas entre motoristas de caminhão, enquanto Sinagawa et al^18^ observaram que motoristas de caminhão que cumpriam trajetórias superiores a 270 km tinham 9,41 mais chances de usar anfetaminas que os pares que percorriam distâncias menores. Entretanto, esses autores não controlaram os efeitos de saúde mental sobre o uso de anfetaminas e não contextualizaram os achados com o decreto que atualmente regulamenta a produção de anfetaminas e tampouco com a legislação que disciplina a atividade dos motoristas profissionais de carga. Entretanto, em conclusão, todos esses dados corroboram o uso de anfetaminas entre os motoristas de caminhão com fins de aumentar a vigília e necessidade de percorrer maiores distâncias, como já previamente descrito.^9^ ^,^ ^11^

Ainda, dirigir por mais de quatro horas ininterruptas também permaneceu como preditor do uso de anfetaminas, porém, com diferença marginalmente significante (p = 0,073). Somado ao achado descrito acima, essas informações apontam para a necessidade de observância dos pressupostos da Lei 12.619/2012, a Lei do Descanso.^b^ Essa lei assegura ao motorista intervalo mínimo de uma hora para refeição, além de intervalo de repouso diário de 11 horas, o que sugere um período de trabalho diário de até 12 horas. Logo, os motoristas que dirigem por mais de 12 horas diárias, além de estarem em desacordo com a lei, estão mais predispostos a usar anfetaminas. Esse dado é consistente com o estudo de Williamson^22^ que mostrou que motoristas de caminhão que usam estimulantes desobedecem as leis de trânsito, especialmente aquelas relacionadas às horas de trabalho. A periculosidade dessa infração é observada pelo fato de que motoristas que dirigem mais de 12 horas diárias estão mais propensos à fadiga e aos seus desdobramentos.^2^ Assim, van der Beek^2^ propôs que a redução do número de horas trabalhadas seja uma das estratégias de proteção à segurança e saúde dos motoristas de caminhão, aumentando as chances de sua adequada recuperação após um dia típico de trabalho e, por outro lado, reduzindo a chance de seu envolvimento em acidentes de trânsito.

Ser autônomo ou trabalhar em turno noturno ou irregular aumentou o risco de uso de anfetaminas. De fato, conforme apontado por Moreno et al,^12^ os motoristas autônomos são mais dependentes da demanda de trabalho, envolvendo-se comumente em fretes de longa distância, engajando-se, para isso, em turnos irregulares de trabalho. E os motoristas que trabalham em turnos irregulares são os que consomem medicamentos estimulantes com maior frequência.^16^ Assim, essa é uma evidência a mais a favor da fiscalização da carga horária de trabalho de motoristas de caminhão no Brasil. A Lei 12.619/2012^b^ atualmente só regulamenta a atividade de motoristas profissionais mediante vínculo empregatício, de tal forma que aqueles que trabalham como autônomos não são fiscalizados.

O uso de anfetaminas entre motoristas de caminhão aumenta em 78,0% a chance de desenvolvimento de comportamentos de risco no trânsito, o que pode ser fatal.^5^ Brodie et al^3^ apontaram que um a cada seis motoristas de caminhão, mortos em acidentes de trânsito, tinham vestígios de anfetaminas em amostras de sangue. Assim sendo, o uso de anfetaminas por condutores é um fator de risco muito importante e que pode estar associado a acidentes graves, inclusive com morte desses profissionais, e a desfechos irreversíveis a terceiros, impactando negativamente a sociedade e os gastos públicos.

Nesse cenário, é preocupante a aprovação do Decreto Legislativo 273/2014,^d^ que então sustou a Resolução 52/2011 da Anvisa,^a^ que proibia a produção e o uso das anfetaminas. Embora haja um longo percurso até a liberação efetiva dessas substâncias no mercado, isso facilitará seu alcance pelos motoristas de caminhão, carregando consigo todos os riscos associados. Entre 2011 e 2014, relatos informais já indicavam que esses profissionais vinham adotando outros estimulantes (a exemplo da cocaína) como substitutos às anfetaminas, de tal forma que o retorno a elas vem a somar comorbidades ao quadro psiquiátrico desses profissionais.

Além de atentar para o controle da venda das anfetaminas, as autoridades públicas devem fiscalizar a lei que regulamenta a organização da atividade ocupacional dos motoristas de caminhão e criar condições adequadas para o cumprimento da Lei 12.619/2012.^b^ Nesse sentido, pode-se estabelecer maior número de locais seguros que possibilitem a parada e descanso dos motoristas de caminhão, de tal forma que tenham condições de desempenhar um trabalho digno, como preconizado pela Organização Internacional do Trabalho.^e^ Ainda, dado o uso múltiplo de drogas entre eles, políticas de combate ao uso de álcool podem ter efeitos sobre o uso de anfetaminas e vice-versa.

Em conclusão, deve ser incentivado o reconhecimento precoce dos motoristas que estejam usando anfetaminas e outras drogas. É também necessário desenvolver campanhas educativas que promovam conhecimentos específicos a essa população. Os motoristas que já estejam sofrendo problemas por conta do uso de anfetaminas devem ser encaminhados a programas especializados para tratamento medicamentoso, aconselhamento psicológico e reabilitação, com consequente vistas à diminuição de reincidência e melhoria da segurança no trânsito.^1^

Essa pesquisa reflete apenas um recorte da situação. O uso de amostra não-probabilística é outro fator limitante, pois os dados não podem ser generalizados à toda população de motoristas de caminhão do Brasil.

## Footnotes

a Ministério da Saúde. Agência Nacional de Vigilância Sanitária. Resolução - RDC nº 52, de 6 de outubro de 2011. Dispõe sobre a proibição do uso das substâncias anfepramona, femproporex e mazindol, seus sais e isômeros, bem como intermediários e medidas de controle da prescrição e dispensação de medicamentos que contenham a substância sibutramina, seus sais e isômeros, bem como intermediários e dá outras providências. *Diário Oficial União*. 10 out 2011;seção 1:55 [citado 2014 ou 15]. Disponível em: http://bvsms.saude.gov.br/bvs/saudelegis/anvisa/2011/res0052_06_10_2011.html

b Brasil. Lei nº 12.619, de 30 de abril de 2012. Dispõe sobre o exercício da profissão de motorista; altera a Consolidação das Leis do Trabalho - CLT, aprovada pelo Decreto-Lei nº 5.452, de 1º de maio de 1943, e as Leis n^os^ 9.503, de 23 de setembro de 1997, 10.233, de 5 de junho de 2001, 11.079, de 30 de dezembro de 2004, e 12.023, de 27 de agosto de 2009, para regular e disciplinar a jornada de trabalho e o tempo de direção do motorista profissional; e dá outras providências. *Diário Oficial União*. 5 maio 2012;seção 1:5 [citado 2014 out 15]. Disponível em: http://www.planalto.gov.br/ccivil_03/_ato2011-2014/2012/lei/l12619.htm

c Universidade Federal de São Paulo, Instituto Nacional de Ciência e Tecnologia para Políticas Públicas de Álcool e Outras Drogas (INPAD). Segundo levantamento nacional de álcool e drogas: relatório 2012. São Paulo (SP): Universidade Federal de São Paulo; 2014.

d Brasil. Decreto Legislativo nº 273, de 2014. Susta a Resolução - RDC nº 52, de 6 de outubro de 2011, da Agência Nacional de Vigilância Sanitária - ANVISA, que dispõe sobre a proibição do uso das substâncias anfepramona, femproporex e mazindol, seus sais e isômeros, bem como intermediários e medidas de controle da prescrição e dispensação de medicamentos que contenham a substância sibutramina, seus sais e isômeros, bem como intermediários. *Diário Oficial União*. 5 set 2014;seção 1:1.

e International Labor Organization (ILO). Decent worg agenda. Geneve: International Labor Organization; 2013 [citado 2014 out 15]. Disponível em: http://www.ilo.org/global/about-the-ilo/decent-work-agenda/lang--en/index.htm
